# A 39 kb structural variant causing Lynch Syndrome detected by optical genome mapping and nanopore sequencing

**DOI:** 10.1038/s41431-023-01494-7

**Published:** 2023-11-29

**Authors:** Pål Marius Bjørnstad, Ragnhild Aaløkken, June Åsheim, Arvind Y. M. Sundaram, Caroline N. Felde, G. Henriette Østby, Marianne Dalland, Wenche Sjursen, Christian Carrizosa, Magnus D. Vigeland, Hanne S. Sorte, Ying Sheng, Sarah L. Ariansen, Eli Marie Grindedal, Gregor D. Gilfillan

**Affiliations:** 1 Department Medical Genetics, Oslo University Hospital and University of Oslo, Oslo, Norway; 2 Department of Clinical & Molecular Medicine, NTNU and Department of Medical Genetics, St Olavs Hospital, Trondheim, Norway; 3 Present Address: Department of Forensic Sciences, Oslo University Hospital, 0372 Oslo, Norway

**Keywords:** Cancer genetics, Cancer genomics, Medical genomics, Genomic analysis, Next-generation sequencing

## Abstract

Lynch Syndrome (LS) is a hereditary cancer syndrome caused by pathogenic germline variants in one of the four mismatch repair (MMR) genes *MLH1*, *MSH2*, *MSH6* and *PMS2*. It is characterized by a significantly increased risk of multiple cancer types, particularly colorectal and endometrial cancer, with autosomal dominant inheritance. Access to precise and sensitive methods for genetic testing is important, as early detection and prevention of cancer is possible when the variant is known. We present here two unrelated Norwegian families with family histories strongly suggestive of LS, where immunohistochemical and microsatellite instability analyses indicated presence of a pathogenic variant in *MSH2*, but targeted exon sequencing and multiplex ligation-dependent probe amplification (MLPA) were negative. Using Bionano optical genome mapping, we detected a 39 kb insertion in the *MSH2* gene. Precise mapping of the insertion breakpoints and inserted sequence was performed by low-coverage whole-genome sequencing with an Oxford Nanopore MinION. The same variant was present in both families, and later found in other families from the same region of Norway, indicative of a founder event. To our knowledge, this is the first diagnosis of LS caused by a structural variant using these technologies. We suggest that structural variant detection be performed when LS is suspected but not confirmed with first-tier standard genetic testing.

## Introduction

On average, humans harbour an estimated 4400 structural variants (SV, genomic alterations of 50 bp or larger) totalling 18 Mb per diploid genome, which are an important underlying cause of genetic disease [1]. However, SV can be difficult to detect. Current genetic diagnostic workflows rely largely on short-read based whole-genome or exome-sequencing, which efficiently and economically detect single-nucleotide variants and short insertions/deletions. In contrast, detection of SV is challenging with short-read technology, and may suffer from low sensitivity and a high false-positive rate [2]. For example, it has been estimated that over 80% of insertions are missed by reliance on short-read sequencing [3]. Conversely, large SV over 50 kb in size are routinely detected by diagnostic array-hybridization platforms [4] or karyotyping. Current diagnostic workflows therefore have significant shortcomings for detection of mid-size SV (approximately 0.05–50 kb).

Long-read sequencing technologies have the potential to bridge this detection gap and have been used to detect SV in a number of studies [5]. However, at present, long-read sequencing technologies do not always match the cost, throughput or accuracy of short-read sequencing (or a combination of these factors), thus have not been widely adopted for routine genetic diagnostics.

An alternative technology for SV detection which relies on high-resolution genome imaging, also known as optical genome mapping (OGM), is well suited for the detection of SV [6, 7]. OGM may offer some advantages for SV detection relative to long-read sequencing due to the focus on maintaining long molecules [8]. However, OGM does not offer the resolution of sequencing, thus alone is unable to determine the precise location of breakpoints. Furthermore, it has limited power to detect short SV (ca. 1 kb and below), and is currently not amenable to high throughput analysis of multiple samples.

Lynch Syndrome is a hereditary cancer syndrome caused by pathogenic germline variants in any of the four DNA mismatch repair (MMR) genes *MLH1*, *MSH2, MSH6* and *PMS2* [9]. Deletions in the 3´end of the upstream *EPCAM* gene have also been shown to cause LS by inactivation of the *MSH2* promoter [10]. LS is the most common hereditary cause of colorectal and endometrial cancers, but pathogenic variant carriers are also at increased risk of developing ovarian, prostate, and ureteral cancers [11]. Prevalence has been estimated to be as high as 1/279 individuals [12]. However, prevalence varies with population sampled, and founder variants present in distinct populations have led to even higher incidences [11, 13]. Identifying the causative variant is important in affected families as at-risk individuals can undergo regular screening to allow early diagnosis and prevention of cancer [14].

Genetic testing of the MMR genes is performed by Sanger-sequencing, or increasingly through the use of multi-gene panels and next generation sequencing [11]. MLPA analysis is also commonly employed to detect insertions/deletions of exonic sequence that could otherwise remain undetected by sequencing approaches alone [15]. Analyses of tumour tissue for aberrant MMR protein expression by immunohistochemistry and/or microsatellite instability (MSI-high), can identify pathogenic MMR variant carriers with a sensitivity of over 73% [15–17]. Prior to the advent of next generation sequencing, these functional analyses were used to select individuals who had a higher likelihood of carrying a pathogenic variant, and therefore should be offered testing.

In this study, we performed extended genetic analysis of two unrelated patients with a personal and family history of cancer consistent with LS. Tumours from both patients demonstrated microsatellite instability and showed lack of MSH2 and MSH6 protein expression by immunohistochemistry, strongly indicative of an underlying pathogenic germline variant in *MSH2* and LS. However, DNA sequencing (both Sanger sequencing of the exons of the above genes and a custom NGS-cancer panel) and MLPA to detect exonic deletions or duplications, failed to reveal a genetic cause to support this diagnosis. Analysis of RNA from cultured patient lymphocytes also did not indicate any aberrant transcripts from the *MSH2* or *MSH6* genes that could indicate a splicing defect. Such patients are in some clinics defined as having Lynch-Like Syndrome [18].

Hypothesizing that the patients could harbour structural genomic variants undetected by the standard tests, we applied Bionano OGM. This revealed a 39 kb heterozygous insertion in the *MSH2* gene, present in both patients. The insertion sequence was determined to be a duplication originating from the neighbouring *MSH6* gene, which lies approximately 250 kb proximal to *MSH2*. This finding was further confirmed using Oxford Nanopore sequencing, which produced reads spanning the entire insertion and led to precise identification of the breakpoints.

## Materials and methods

### Patient selection

Two families for which we had a strong suspicion of LS based on family history and immunohistochemistry/MSI results, but where we had been unable to find a causative MMR variant with techniques available in our diagnostic laboratory, were selected for this study.

The index patient in family 1 had been diagnosed with colorectal cancer in his 50s and prostate cancer in his 60s. His family history fulfilled the Amsterdam Criteria used to select individuals for genetic testing for LS [19]. Immunohistochemistry of his prostate tumour showed lack of staining of MSH2 and MSH6 and the tumour was MSI-high. The analyses were also performed on tumour tissue from a paternal aunt and her daughter with the same results. No variants were identified by Sanger sequencing, MLPA or cDNA-analysis of *MSH2* (all 16 exons of transcript variant NM_000251.1).

The index patient in family 2 was diagnosed with colorectal and prostate cancers in his 40s. His family history of cancer also fulfilled the Amsterdam criteria. Immunohistochemistry and MSI analyses were performed on his colorectal tumour, and on tumour tissue from three other cancers in his first-degree relatives. All tumours lacked staining of MSH2 and MSH6 and were MSI-high. Sanger sequencing, MLPA and cDNA-analysis revealed no variants in *MSH2* and only benign variants in *MSH6*. The absence of exonic variants precluded the possibility to examine mono/bi-allelic expression of *MSH2* by cDNA analysis in the presence/absence of puromycin, for example as described previously [20].

### Immunohistochemistry

Paraffin sections (3 µm) were stained on a BenchMark Ultra instrument (Roche, Basel, Switzerland). For antigen retrieval the slides were pre-treated in Cell Conditioner 1 (Roche) for 64 min for MLH1, MSH2, MSH6 and 92 min for PMS2. Signals were detected with OptiView DAB IHC Detection Kit (Roche). For MLH1 and PMS2, OptiView Amplification Kit (Roche) was used in addition. Primary antibodies were purchased from Roche, and the incubation time for MLH1 (clone M1, 760-5091), MSH2 (clone G219-1129, 760-5093) and PMS2 (clone A16-4, 760-5094) was 32 min, and for MSH6 (clone SP93, 760-5092) 12 min. Chromogene incubation time was 8 min, with subsequent CuSO_4_ intensification for 4 min, with the slides then contrast-stained with haematoxylin.

### Optical genome mapping

For each sample, 650 µL of whole peripheral blood was used to purify ultra-high molecular weight (UHMW) DNA using the SP Blood & Cell Culture DNA Isolation Kit v2 (Bionano genomics, San Diego, CA, USA), following the manufacturer´s instructions for frozen human blood. UHMW DNA (750 ng) was then labelled using the DLS (Direct Label and Stain) DNA Labelling Kit (Bionano genomics), following protocol revision F. 19.5 µL of labelled UHMW DNA solution of concentration 4 -12ng/µl was loaded on a Saphyr chip and scanned on the Saphyr instrument (Bionano genomics), following Saphyr System User Guide revision D. The Saphyr chip was run targeting 320 Gb data per sample (100X coverage), yielding 500 Gb data. All samples met the recommended metrics (effective coverage, map rate, N50, label density). After default filtering, mean molecule length was 251 kb, median 224 kb and N50 was 255 kb.

De novo assembly was executed on Bionano Solve software V3.7. Reporting and direct visualization of SV was done on Bionano Access V1.7.0. Recommended filtering was used corresponding to the following minimum confidence values: insertion/deletion=0, inversion=0.7, duplications = -1, intra-fusion/inter-translocation=0.05, CNV = 0.99, and aneuploidy= 0.95. Events detected were subsequently filtered against a normal samples database and only variants absent (or present at a percentage below 1%) of that database were considered for analysis. DLE-1 SV masked regions and CNV masked regions were filtered out.

### MinION sequencing

UHMW gDNA isolated for the Bionano analysis was also used for MinION sequencing. 2 µg DNA was partially sheared by 20 passages through a 25-gauge needle. Libraries were prepared for sequencing using the Genomic DNA by Ligation (SQK-LSK109) kit (Oxford Nanopore Technologies, Oxford, UK), according to the manufacturer’s instructions. Sequencing was performed by loading 25 fmol library on a MinION Mk1c instrument with flowcell version 9.4.1. Basecalling was performed using Guppy v6.0.1 in high-accuracy mode. Reads had a mean length of 19 kb, median 6 kb and N50 was 58 kb. Reads were aligned to the GRCh38 reference genome using Minimap2 v2.24 [21]. The position of the insertion was roughly identified by manual inspection in IGV [22], and the reads spanning this point were extracted using samtools [23]. The reads were then analysed using BLAT hosted at the UCSC genome browser [24] to find the breakpoints and the contents of the insertion.

### Breakpoint PCR and Sanger Sequencing

Breakpoints were confirmed by PCR amplification with the following primers, designed to amplify from unique sequences flanking the approximate breakpoints identified by MinION sequencing: wt-allele (for. TTGTGCCTCTATTTCTCCATTCTG rev. GGGAGACTTTTCATTTTGTTCTGTACTA); left-ins (for. TGTCTTCCACTGCTGTGCTTTTCT rev. GAGTCTGGCTTTGGCTTTGTCAC); right-ins (for. AGTTAATTTGCGGGCCCCTGAT rev. AGGGTTGAACGGATTAAGGGT). Primers were designed with M13-tails to prime subsequent sequencing reactions. M13 sequences were as follows: M13-forward primer; TGTAAAACGACGGCCAGT and M13-reverse primer; CAGGAAACAGCTATGACC. PCR was performed with AmpliTaq Gold™ 360 DNA Polymerase (Applied Biosystems, Waltham, MA, USA), with 6.25 ng/µL template DNA, and primers at 0.5 µM with the following conditions: 95 ^o^C for 10 min (denaturation), followed by 30 cycles of 95 ^o^C for 30 s / 60 ^o^C for 30 s / 72 ^o^C for 60 s, with a final extension at 72 ^o^C for 7 min. PCR products were sequenced on an ABI 3730xl DNA analyzer (Applied Biosystems) using the BigDye Terminator v3.1 Cycle Sequencing Kit (Applied Biosystems, Waltham, MA, USA).

### RNA extraction, PCR and sequencing

Patient and control blood samples were collected in PAXgene Blood RNA tubes (PreAnalytiX GmbH, Hombrechtikon, Switzerland). Total RNA was extracted using the PAXgene ® Blood RNA Kit (PreAnalytiX), including an on-column digestion of DNA using RNAse-free DNAse I (Qiagen, Venlo, Netherlands).

Reverse-Transcriptase-PCR and PCR-amplification of the target region was conducted in a one-step-reaction using the OneStep RT-PCR kit (Qiagen), with 150 ng input RNA. The manufacturer’s procedures were followed, with the exception that reactions were run at half the suggested volume. Thermal cycling conditions were 50 ^o^C for 30 min (reverse transcription), 95 ^o^C for 15 min (PCR activation), followed by 35 cycles of 95 ^o^C for 30 s / 60 ^o^C for 30 s / 72 ^o^C for 60 s, with a final extension at 72 ^o^C for 7 min.

Primers were designed to reveal if the *MSH6* exons inserted in the genomic sequence were spliced between *MSH2* exons 7 and 8 of the *MSH2* mRNA. Primer sequences are provided in Table 1. Patient samples were run in duplicate together with four non-carrier control samples. RT-PCR products were sequenced on an ABI 3730xl DNA analyzer (Applied Biosystems) as above.

**Table 1:** RT-PCR primers.

Primer pair	Transcript ID	Refseq Match	Gene	Exon	Primer sequence (5′-3′)
**1**	ENST00000233146.6	NM_000251.2	MSH2	6	Forward : M13 + AGTGGATTAAGCAGCCTCTCA
	ENST00000652107.1	–	MSH6	2	Reverse: M13 + AGGTGAATCACAGCCGATGA
**2**	ENST00000652107.1	–	MSH6	2	Forward: M13 + TCATCGGCTGTGATTCACCT
	ENST00000233146.6	NM_000251.2	MSH2	8	Reverse: M13 + TCCTGAAACTTGGAGAAGTCAGA
**3**	ENST00000652107.1	–	MSH6	1	Forward: M13 + AGCTTCCAGAAGAGCAGAGCT
	ENST00000233146.6	NM_000251.2	MSH2	8	Reverse: M13 + TCCTGAAACTTGGAGAAGTCAGA

Three PCR products (primer pairs 1–3 above) were amplified with primers from *MSH2* and *MSH6* to detect *MSH6* exons inserted in the *MSH2* transcript by Sanger sequencing. M13 sequences were as follows: M13-forward primer; TGTAAAACGACGGCCAGT and M13-reverse primer; CAGGAAACAGCTATGACC.

### Illumina sequencing

DNA was prepared for sequencing using the TruSeq DNA PCR-free paired-end protocol (Illumina, San Diego, CA, USA) and WGS was performed on a NovaSeq 6000 instrument (Illumina) with 150 bp paired end sequencing to approximately 30X coverage with v1.5 reagents. Processing of WGS sequencing data was performed using RTA v3.4.4 and bcl2fastq v2.20.0.422, with variant calling (including both small and structural variants) on Illumina DRAGEN (Dynamic Read Analysis for GENomics) Bio-IT platform, Ensembl Variant Effect Predictor (VEP) and AnnotSV [25]. BAM files were visualized using the Integrative Genomics Viewer (IGV) [26].

### Co-segregation analysis

Co-segregation analysis based on the full-likelihood Bayes factor (FLB) [27] was performed using extended pedigrees of the two families (data not shown), assuming an autosomal dominant model and a population frequency of the variant of 0.1%. To estimate penetrance, we obtained incidence data for any cancer in pathogenic *MSH2* variant carriers from the Prospective Lynch Syndrome Database [14]. A pre-smoothing step was performed to reduce the uncertainty in age-specific incidence rates, by means of Poisson regression with a penalized thin-plate spline (k = 3). To estimate the phenocopy rate in non-carriers, we used data from Cancer Incidence in Five Continents [28] for the Norwegian population (2008-2012). Penetrance values were calculated by sex and 5-year age group following the survival model [29]. Individuals of unknown age were assigned the averages. The FLB was computed via the R package segregatr [30], using the thresholds suggested by Jarvik and Browning [31] for translation to the ACMG-AMP framework.

### Relatedness analysis

Identical by descent (IBD) genomic segments shared by the index patients were inferred from the identity by state (IBS) status of the observed variants in the WGS data using a sliding window approach resembling that used by PLINK [32]. As a preparatory step, a filter was applied, keeping only single-nucleotide variants where both individuals had high-quality genotype calls (PASS; DP > 10; GQ > 50). The IBS state (either 0, 1 or 2) of each variant was recorded, as well as the average occurrence $w$ of IBS = 0 in a neighbourhood ranging 1 Mb upstream and 1 Mb downstream. Runs of at least 100 variants with $w\le 0.5 \%$, spanning at least 1 cM, were interpreted as IBD segments.

## Results

**Fig. 1:**
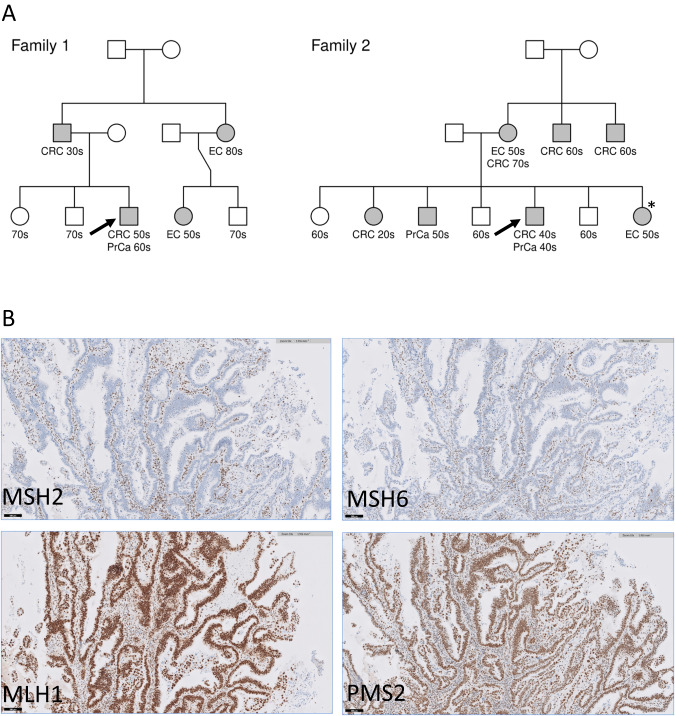
LS-like heritability and MMR protein expression. **A** Pedigrees of families 1 and 2. Cancer type and decade of onset as follows: Colorectal cancer (CRC), prostate cancer (PrCa) and endometrial cancer (EC). Pedigrees have been modified for patient confidentiality. Arrows indicate index-patients selected for Bionano OGM. **B** Immunohistochemistry of patient endometrial tumour biopsy. Immunohistochemical staining of endometrial tumour tissue sections with antibodies against the four MMR proteins (brown staining) showed missing protein expression of MSH2 and MSH6, while normal expression was obtained for MLH1 and PMS2. Slides were co-stained with hematoxylin (blue) to show cell nuclei. Sections were endometrial tumour tissue from the individual marked with an asterisk in Family 2.

Faced with two cases of familial cancer compatible with LS (Fig. 1A) that had returned negative results for MMR variants by Sanger sequencing (DNA and RNA) and MLPA, we decided to screen for possible SV using the Bionano OGM technology. Both cases showed loss of MSH2 and MSH6 protein expression by immunohistochemistry of tumour samples (Fig. 1B), suggesting that MSH2 was inactivated [33].

**Fig. 2:**
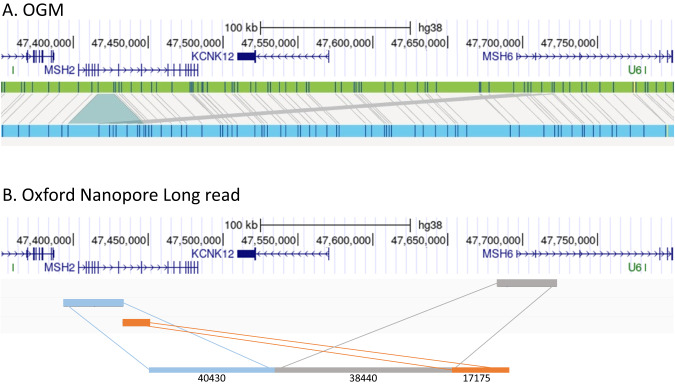
OGM and long-read sequencing. **A** Optical Genome Mapping. Genome-browser view of OGM results, showing ca. 300 kb region encompassing the *MSH2* gene from the index patient in family 2. Scale and co-ordinates from GRCh38 (hg38) genome annotation are shown above gene models. The green bar shows the OGM reference genome markers (blue stripes). Light-blue bar shows OGM assembly from patient DNA. Grey bars matching between reference and patient assembly show the insertion of new sequence, and suggest the origin of inserted sequence to be the neighbouring *MSH6* gene. **B** Oxford Nanopore Long read sequencing. Genome browser view of the same 300 kb region, showing alignment of a single long read (length 96,045 bp) spanning the *MSH2* locus with inserted *MSH6* sequence. The sections of alignment to *MSH2* and *MSH6* are coloured to aid visualization, with approximate alignment lengths in bp below each section.

We hypothesized that in both families an SV could be responsible for disruption of the *MSH2* gene. A single individual (index case) from each family was therefore studied by OGM. In both cases, the Bionano OGM revealed an insertion of ca. 39 kb in the *MSH2* gene (Fig. 2A), raising the possibility that the insertion disrupted the gene, thereby causing LS. Notably, the insertion detected by OGM was strikingly similar in both cases suggesting the two individuals may have inherited the variant from a common ancestor. This theory was supported by the fact that both families came from the same geographical region of Norway. The OGM results also identified the inserted sequence as arising from the nearby *MSH6* locus, which lies ca. 250 kb centromeric to *MSH2* on the p arm of chromosome 2, suggesting that one MMR gene was disrupted by sequence from a second MMR gene (Fig. 2A).

**Fig. 3:**
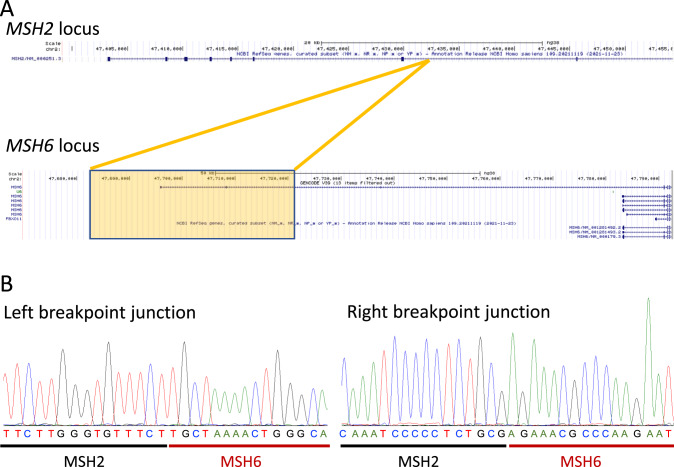
Insertion and breakpoint sequences. **A** UCSC genome browser view of insertion. Upper panel shows *MSH2* locus (NCBI reference sequence NM_000251.3), lower panel the *MSH6* locus. Orange box and lines indicate extent of *MSH6* sequence that is duplicated and insertion point in *MSH2*. Two 5ʹUTR non-coding exons from *MSH6* transcript variant ENST00000652107.1 are thus inserted between exons 7 and 8 of *MSH2*. **B** Sanger sequences of breakpoints. The breakpoint in *MSH2* sequence was determined to be chr2:47,432,457. The inserted sequence duplicated from *MSH6* was chr2:47,682,947–47,721,794 (genomic positions from GRCh38 genome release). The right-hand breakpoint sequence is presented in the reverse complement of the database genomic sequence. The full variant is therefore annotated as NC_000002.12:g.47432456_47432457ins47682947_47721794.

To confirm the identity of the inserted sequence and to determine the insertion breakpoints in *MSH2*, we performed low-coverage WGS with Oxford Nanopore long-read sequencing. Using a single MinION flowcell, we obtained an average 2.8X genome coverage from the index-case individual from family 1. Sequencing depth was insufficient to allow automated SV detection using long-read alignment software LRA or Minimap2 in combination with the SV detection software CuteSV. However, by manually selecting the MinION reads aligning to the *MSH2* locus and employing BLAT [34], we were able to identify the breakpoints in *MSH2* to within ±50 bp. The insertion was thus confirmed to be duplicated sequence derived from the *MSH6* locus (Fig. 2B), duplicating two 5ʹ UTR non-coding exons from a minor transcript variant of *MSH6* (Fig. 3A). The *MSH6* exons were inserted in the intron between exons 7 and 8 of the *MSH2* gene, and if either or both were spliced into the *MSH2* transcript, were predicted to introduce premature stop codons.

Both the insertion point in *MSH2* and the right junction of the *MSH6* insertion occurred in homologous repetitive sequences derived from the SINE-VNTR-Alu (SVA) retrotransposon [35]. At 2.1 kb, the SVA repeat at *MSH2* is potentially a full-length functional repeat, whilst the *MSH6* SVA sequence is a short (265 bp) remnant. However, transposition was not suspected to be the mutagenic event in this instance, as no new insertion of SVA sequence was detected. Rather, a recombination event was deemed more likely. The left-hand junction of the inserted sequence from *MSH6* was determined to lie within a 240 bp LTR sequence derived from an endogenous retrovirus (ERV1 family) [36].

Based on the approximate breakpoint positions identified by long-read sequencing, PCR primers were designed to amplify the breakpoints, providing unequivocal confirmation of the insertion sequence. Sanger sequencing of the amplified fragments confirmed the precise breakpoints (Fig. 3B). The same PCR reactions were then used to offer cascade-testing to additional family members in both families (Supplementary Fig. 1).

**Fig. 4:**
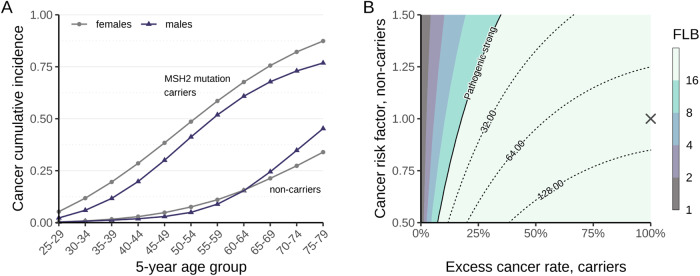
Co-segregation analysis. **A** Cumulative incidence for any cancer by sex, age-group and carriership status. Data for *MSH2* mutations carriers from the Prospective Lynch Syndrome Database [14] (smoothed), and data for non-carriers from Cancer Incidence in Five Continents [28], Norway 2008–2012. **B** Co-segregation sensitivity analysis, showing the contours of the FLB factor as a function of the risk of cancer in non-carriers and the excess rate in carriers. The robustness of the results was evaluated by recomputing the FLB under a range of ever-more conservative penetrance parameters: we varied the cumulative incidences in non-carriers by a factor of 0.50–1.50 and, for each value of this factor, the incidence rates of *MSH2* carriers were also varied as a weighted average between the non-carrier incidence rates and their original estimates. These endpoints are represented as 0–100% excess cancer rates, respectively. The cross corresponds to the main co-segregation result reported in the study. The contour curve at FLB = 16 signifies the threshold for pathogenic strong evidence according to Jarvik and Browning [31].

Bayes factor-based co-segregation analysis provided strong evidence for pathogenicity, according to the full-likelihood Bayes factor (FLB) thresholds suggested by Jarvik & Browning [31]. The individual FLB scores for the two families were 12.10 and 8.13, respectively, both exceeding the threshold for pathogenic supporting evidence (FLB > 8). Furthermore, the combined score (FLB = 98.40) comfortably satisfied the criterion at the pathogenic strong level (FLB > 16). Importantly, this conclusion remained robust across a wide range of more conservative penetrance values (Fig. 4).

To further substantiate the insertion as the underlying cause of LS in the affected families, RNA was extracted from a blood sample (family 2, index case). RNA was reverse-transcribed to cDNA, then amplified with PCR primers spanning the possible exon-junctions. The second *MSH6* exon was detected in the *MSH2* transcript (Supplementary Fig. 2), leading to the inclusion of a premature stop codon in the resulting transcript. A minor variant also containing part of the first *MSH6* exon, plus the full second exon, was also detected. This variant also led to the inclusion of a premature stop codon.

Short read WGS was also performed for the index case from both families (Illumina 150 bp paired end sequencing) to median 45x and 49x genome coverage. To examine whether variant calling using the short-read data could detect the SV, we employed the DRAGEN SV Caller. However, in one case the caller failed to detect an SV in either *MSH2* or *MSH6*, and in the other the variant was incorrectly detected as a 250 kb deletion, spanning the approximate *MSH2* breakpoint to the start of the *MSH6* gene. The duplicated region of *MSH6* was, however, called by our read-depth CNV caller (canvas), but filtered out from the final CNV results due to lack of overlap with our gene panel *MSH6* transcript. Our short-read diagnostic pipeline therefore did not correctly report the variant, but with the benefit of knowing the SV location from the Bionano & long-read analyses, our in-house database of WGS-CNV data could be screened for the presence of the duplicated region. The duplicated region was not detected in our WGS-CNV database of 7710 individuals, confirming that this is a rare variant, not a common Nordic variant. Based on these observations the variant was categorized as pathogenic according to ACMG guidelines [37] and reported to be a likely cause of LS in the affected families.

Finally, we examined the relatedness of the two affected families to estimate the number of generations that had passed since the shared mutation occurred. Regions of identity by descent (IBD) between the two index cases were inferred from the WGS data, using a sliding-window approach along each chromosome. Nine IBD regions over 1 cM in length were identified (Supplementary Fig. 3), the majority being short sections unlikely to originate from a recent common ancestor. The longest segment, a 17 cM region on chromosome 2, encompassed the *MSH2* and *MSH6* genes. From a single such IBD segment it is inherently difficult to give an accurate estimate on the relationship between the individuals. However, comparing with theoretical distributions [38] a segment of 17 cM indicates that they most likely shared a common ancestor 4–6 generations prior.

## Discussion

Current techniques available for routine genetic analyses, such as Sanger sequencing, aCGH, MLPA and short-read exome/WGS sequencing, have shortcomings when it comes to SV detection. Insertions, deletions or rearrangements are frequently missed due to limitations in resolution, particularly if repetitive elements are involved, combined with the focus on coding exon sequences. With over half of the human genome consisting of dispersed repetitive elements [39], compounded by the increased likelihood of recombination events occurring between repetitive sequences [40, 41], current practices will thus likely miss many pathogenic SV. The SV found in this study is a good example of such an event, consisting of inserted non-coding sequence with breakpoints in repetitive elements.

By the application of OGM, we were able to detect an SV causing LS by heterozygous disruption of the *MSH2* gene. The 39 kb insertion placed non-coding 5´UTR exons from *MSH6* into intron 7 of the *MSH2* gene. mRNA studies confirmed that a non-coding exon was spliced into *MSH2* RNA, creating a truncated protein and causing loss of function. The variant segregated with several individuals from the two different families showing loss of MSH2 and MSH6 protein expression and MSI-high tumours. For these reasons the variant has been classified as pathogenic. This finding demonstrates the advantages of OGM over routine diagnostic methods for SV detection. At 39 kb, the SV found in this study is well above the lower resolution claimed for OGM (ca. 0.5 kb). However, despite the inclusion of only seven OGM markers, the technique was able to correctly identify the source of the inserted sequence, underlining its utility. In this case, the finding confirmed the diagnosis of LS in two families that had been uncertain for around 15 years, and enabled relatives to obtain genetic testing and clarification of their cancer risk.

Nonetheless, this study also demonstrates a limitation of OGM, which does not have the resolution to determine breakpoints to within the distances required to design PCR primers for verification. We therefore employed also long-read sequencing with Oxford Nanopore technology to verify the OGM findings and improve detection of the breakpoints themselves. Although the data from a single MinION flowcell on this occasion confirmed the insertion sequence and position, the low coverage (2.8-fold genome coverage) prevented long-read variant calling software from identifying the sequences, and manual inspection of BLAST alignments was necessary. The low coverage and high error rate of the MinION reads also meant we were unable to precisely identify the breakpoints. Nonetheless, the long reads were instrumental in this regard, allowing breakpoint identification to within ±50 bp, and facilitating design of PCR primers that allowed breakpoint identification by Sanger sequencing.

It is likely that deeper sequencing with long reads (for example with higher-output PromethION flowcells), or the use of targeted-capture combined with MinION sequencing (as has been demonstrated for *MSH2* [42]) would be more effective than low-coverage WGS MinION sequencing for breakpoint detection. Indeed, WGS approaches may on their own suffice to identify SV without the prior need for optical mapping. However, these approaches also cost more, so for the purposes of verifying Bionano optical genome mapping findings, Oxford Nanopore’s recently released adaptive sampling method, employed with a MinION flowcell, may offer the most cost-effective approach.

Dispersed repeats such as the SVA and LTR elements found at the breakpoints of the insertion in this study can contribute to pathogenic SV through either transposition or recombination events [35]. The SVA repeats (SINE-VNTR-Alu) are non-LTR retrotransposons, similar to LINE-1 and Alu elements. As their name suggests, SVA retrotransposons are composite elements consisting of sequences derived from each of the contributing retrotransposons. However, unlike LINE-1 and Alu elements, which have proliferated widely throughout the human genome to constitute approximately 17% and 10% of the human genome respectively [40], SVA elements are relatively rare: It is estimated that there are only approx. 2700 SVA copies in the human genome (ca. 0.2%) [43].

SVA repeats have been shown to be involved in pathogenic SV formation [35]. More specifically, SVA transposition events in *MSH2, MSH6* and *PMS2*, have been reported to cause LS [44–46]. In the patients reported in the present study, the *MSH2* insertion point and donor *MSH6* site contain the SVA_D_ retrotransposon repeat element (SINE-VNTR-Alu, subfamily D) [35]. The SVA_D_ copy at the *MSH2* insertion site appears to be a full-length element, while a truncated fragment is found at the *MSH6* donor site. There is no evidence suggesting an SVA transposition event, but rather the contribution of existing SVA repeats to the generation of an SV through recombination. Since the left-hand side of the duplicated *MSH6* sequence contains sequence from the unrelated ERV1 LTR repeat, a complex recombination event is assumed to have occurred.

The 39 kb insertion detected in both families, who were not aware they were related, was determined to have originated from a founding mutation event estimated to have occurred at least 4 generations prior to the two index cases presented here. Analysis of additional individuals will be required to more accurately estimate the timing of the founder event, which could have occurred in the more distant past. At the time of writing, individuals from three additional families (none aware of their relation to the others), also from the same region of Norway and presenting with suspected LS, have since been confirmed to carry the variant. In all these families, immunohistochemistry demonstrated no expression of MSH2 and MSH6 in tumour tissue, and their cancers were MSI-high.

It has been recommended for over a decade that immunohistochemistry and MSI analyses be performed on patients with colorectal or endometrial cancer [47]. These methods, which can indicate with up to 90% sensitivity an underlying MMR variant, can be used to prioritize patients for extended genetic testing, including for SV. SV are a common source of pathogenic variants in the LS genes. Some of these, but not all, are detectable through MLPA or exome sequencing [42, 48–50]. We therefore encourage the use of OGM or long-read sequencing technologies to test for the presence of SV variants where LS is suspected based on family history and/or immunohistochemistry/MSI findings but a genetic diagnosis has not been obtained with routine analyses. The recent observation that cancer-associated genes are enriched for retro-elements, which may act as recombination hotspots [51], supports the increased use of techniques such as OGM or long-read sequencing to evaluate SV as causative variants also in other familial cancers.

### Supplementary information

Supplementary Figures

## Data Availability

The datasets generated during and/or analysed during the current study are not publicly available due to patient confidentiality but are available from the corresponding author on reasonable request.

## References

[1] Sudmant PH, Rausch T, Gardner EJ, Handsaker RE, Abyzov A, Huddleston J (2015). An integrated map of structural variation in 2504 human genomes. Nature.

[2] Mahmoud M, Gobet N, Cruz-Davalos DI, Mounier N, Dessimoz C, Sedlazeck FJ (2019). Structural variant calling: the long and the short of it. Genome Biol.

[3] Chaisson MJP, Sanders AD, Zhao X, Malhotra A, Porubsky D, Rausch T (2019). Multi-platform discovery of haplotype-resolved structural variation in human genomes. Nat Commun.

[4] Coe BP, Ylstra B, Carvalho B, Meijer GA, Macaulay C, Lam WL (2007). Resolving the resolution of array CGH. Genomics.

[5] Logsdon GA, Vollger MR, Eichler EE (2020). Long-read human genome sequencing and its applications. Nat Rev Genet.

[6] Barseghyan H, Tang W, Wang RT, Almalvez M, Segura E, Bramble MS (2017). Next-generation mapping: a novel approach for detection of pathogenic structural variants with a potential utility in clinical diagnosis. Genome Med.

[7] Lam ET, Hastie A, Lin C, Ehrlich D, Das SK, Austin MD (2012). Genome mapping on nanochannel arrays for structural variation analysis and sequence assembly. Nat Biotechnol.

[8] Ebert P, Audano PA, Zhu Q, Rodriguez-Martin B, Porubsky D, Bonder MJ, et al. Haplotype-resolved diverse human genomes and integrated analysis of structural variation. Science. 2021;372:eabf7117. 10.1126/science.abf7117 PMC8026704 33632895

[9] Nolano A, Medugno A, Trombetti S, Liccardo R, De Rosa M, Izzo P, et al. Hereditary Colorectal Cancer: State of the Art in Lynch Syndrome. Cancers (Basel). 2022;15:75. 10.3390/cancers15010075 PMC9817772 36612072

[10] Ligtenberg MJ, Kuiper RP, Chan TL, Goossens M, Hebeda KM, Voorendt M (2009). Heritable somatic methylation and inactivation of MSH2 in families with Lynch syndrome due to deletion of the 3′ exons of TACSTD1. Nat Genet.

[11] Biller LH, Syngal S, Yurgelun MB (2019). Recent advances in Lynch syndrome. Fam Cancer.

[12] Win AK, Jenkins MA, Dowty JG, Antoniou AC, Lee A, Giles GG (2017). Prevalence and penetrance of major genes and polygenes for colorectal cancer. Cancer Epidemiol Biomark Prev.

[13] Haraldsdottir S, Rafnar T, Frankel WL, Einarsdottir S, Sigurdsson A, Hampel H (2017). Comprehensive population-wide analysis of Lynch syndrome in Iceland reveals founder mutations in MSH6 and PMS2. Nat Commun.

[14] Dominguez-Valentin M, Sampson JR, Seppala TT, Ten Broeke SW, Plazzer JP, Nakken S (2020). Cancer risks by gene, age, and gender in 6350 carriers of pathogenic mismatch repair variants: findings from the Prospective Lynch Syndrome Database. Genet Med.

[15] Snowsill T, Huxley N, Hoyle M, Jones-Hughes T, Coelho H, Cooper C (2014). A systematic review and economic evaluation of diagnostic strategies for Lynch syndrome. Health Technol Assess.

[16] Evrard C, Tachon G, Randrian V, Karayan-Tapon L, Tougeron D. Microsatellite instability: diagnosis, heterogeneity, discordance, and clinical impact in colorectal cancer. Cancers (Basel). 2019;11:1567. 10.3390/cancers11101567 PMC6826728 31618962

[17] Snowsill T, Coelho H, Huxley N, Jones-Hughes T, Briscoe S, Frayling IM (2017). Molecular testing for Lynch syndrome in people with colorectal cancer: systematic reviews and economic evaluation. Health Technol Assess.

[18] Martinez-Roca A, Giner-Calabuig M, Murcia O, Castillejo A, Soto JL, Garcia-Heredia A, et al. Lynch-like syndrome: potential mechanisms and management. Cancers (Basel). 2022;14:1115. 10.3390/cancers14051115 PMC8909420 35267422

[19] Vasen HF, Watson P, Mecklin JP, Lynch HT (1999). New clinical criteria for hereditary nonpolyposis colorectal cancer (HNPCC, Lynch syndrome) proposed by the International Collaborative group on HNPCC. Gastroenterology.

[20] Morak M, Schaefer K, Steinke-Lange V, Koehler U, Keinath S, Massdorf T (2019). Full-length transcript amplification and sequencing as universal method to test mRNA integrity and biallelic expression in mismatch repair genes. Eur J Hum Genet.

[21] Li H (2021). New strategies to improve minimap2 alignment accuracy. Bioinformatics.

[22] Robinson JT, Thorvaldsdottir H, Winckler W, Guttman M, Lander ES, Getz G (2011). Integrative genomics viewer. Nat Biotechnol.

[23] Danecek P, Bonfield JK, Liddle J, Marshall J, Ohan V, Pollard MO, et al. Twelve years of SAMtools and BCFtools. Gigascience. 2021;10:giab008. 10.1093/gigascience/giab008 PMC7931819 33590861

[24] Kent WJ, Sugnet CW, Furey TS, Roskin KM, Pringle TH, Zahler AM (2002). The human genome browser at UCSC. Genome Res.

[25] Geoffroy V, Guignard T, Kress A, Gaillard JB, Solli-Nowlan T, Schalk A (2021). AnnotSV and knotAnnotSV: a web server for human structural variations annotations, ranking and analysis. Nucleic Acids Res.

[26] Thorvaldsdottir H, Robinson JT, Mesirov JP (2013). Integrative Genomics Viewer (IGV): high-performance genomics data visualization and exploration. Brief Bioinform.

[27] Thompson D, Easton DF, Goldgar DE (2003). A full-likelihood method for the evaluation of causality of sequence variants from family data. Am J Hum Genet.

[28] Bray FCM, Mery L, Piñeros M, Znaor A, Zanetti R, Ferlay J. Cancer Incidence in Five Continents Lyon: International Agency for Research on Cancer, (2017), Vol. XI (electronic version).

[29] Belman S, Parsons MT, Spurdle AB, Goldgar DE, Feng BJ (2020). Considerations in assessing germline variant pathogenicity using cosegregation analysis. Genet Med.

[30] Ratajska A, Vigeland MD, Wirgenes KV, Krohg-Sorensen K, Paus B (2023). The use of segregation analysis in interpretation of sequence variants in SMAD3: A case report. Mol Genet Genom Med.

[31] Jarvik GP, Browning BL (2016). Consideration of cosegregation in the pathogenicity classification of genomic variants. Am J Hum Genet.

[32] Purcell S, Neale B, Todd-Brown K, Thomas L, Ferreira MA, Bender D (2007). PLINK: a tool set for whole-genome association and population-based linkage analyses. Am J Hum Genet.

[33] Pearlman R, Markow M, Knight D, Chen W, Arnold CA, Pritchard CC (2018). Two-stain immunohistochemical screening for Lynch syndrome in colorectal cancer may fail to detect mismatch repair deficiency. Mod Pathol.

[34] Kent WJ (2002). BLAT-the BLAST-like alignment tool. Genome Res.

[35] Hancks DC, Kazazian HH (2010). SVA retrotransposons: Evolution and genetic instability. Semin Cancer Biol.

[36] Vargiu L, Rodriguez-Tome P, Sperber GO, Cadeddu M, Grandi N, Blikstad V (2016). Classification and characterization of human endogenous retroviruses; mosaic forms are common. Retrovirology.

[37] Richards S, Aziz N, Bale S, Bick D, Das S, Gastier-Foster J (2015). Standards and guidelines for the interpretation of sequence variants: a joint consensus recommendation of the American College of Medical Genetics and Genomics and the Association for Molecular Pathology. Genet Med.

[38] Speed D, Balding DJ (2015). Relatedness in the post-genomic era: is it still useful?. Nat Rev Genet.

[39] de Koning AP, Gu W, Castoe TA, Batzer MA, Pollock DD (2011). Repetitive elements may comprise over two-thirds of the human genome. PLoS Genet.

[40] Cordaux R, Batzer MA (2009). The impact of retrotransposons on human genome evolution. Nat Rev Genet.

[41] Bourque G, Burns KH, Gehring M, Gorbunova V, Seluanov A, Hammell M (2018). Ten things you should know about transposable elements. Genome Biol.

[42] Yamaguchi K, Kasajima R, Takane K, Hatakeyama S, Shimizu E, Yamaguchi R (2021). Application of targeted nanopore sequencing for the screening and determination of structural variants in patients with Lynch syndrome. J Hum Genet.

[43] Wang H, Xing J, Grover D, Hedges DJ, Han K, Walker JA (2005). SVA elements: a hominid-specific retroposon family. J Mol Biol.

[44] Yamamoto G, Miyabe I, Tanaka K, Kakuta M, Watanabe M, Kawakami S (2021). SVA retrotransposon insertion in exon of MMR genes results in aberrant RNA splicing and causes Lynch syndrome. Eur J Hum Genet.

[45] van der Klift HM, Tops CM, Hes FJ, Devilee P, Wijnen JT (2012). Insertion of an SVA element, a nonautonomous retrotransposon, in PMS2 intron 7 as a novel cause of Lynch syndrome. Hum Mutat.

[46] Yang C, Li Y, Trottier M, Farrell MP, Rai VK, Salo-Mullen EE (2021). Insertion of an SVA element in MSH2 as a novel cause of Lynch syndrome. Genes Chromosomes Cancer.

[47] Evaluation of Genomic Applications in P, Prevention Working G. (2009). Recommendations from the EGAPP Working Group: genetic testing strategies in newly diagnosed individuals with colorectal cancer aimed at reducing morbidity and mortality from Lynch syndrome in relatives. Genet Med.

[48] Lagerstedt-Robinson K, Rohlin A, Aravidis C, Melin B, Nordling M, Stenmark-Askmalm M (2016). Mismatch repair gene mutation spectrum in the Swedish Lynch syndrome population. Oncol Rep..

[49] Baudhuin LM, Ferber MJ, Winters JL, Steenblock KJ, Swanson RL, French AJ (2005). Characterization of hMLH1 and hMSH2 gene dosage alterations in Lynch syndrome patients. Gastroenterology.

[50] Mu W, Li B, Wu S, Chen J, Sain D, Xu D (2019). Detection of structural variation using target captured next-generation sequencing data for genetic diagnostic testing. Genet Med.

[51] Pascarella G, Hon CC, Hashimoto K, Busch A, Luginbuhl J, Parr C (2022). Recombination of repeat elements generates somatic complexity in human genomes. Cell.

