# Publisher Correction: A 39 kb structural variant causing Lynch syndrome detected by optical genome mapping and nanopore sequencing

**DOI:** 10.1038/s41431-023-01519-1

**Published:** 2024-01-04

**Authors:** Pål Marius Bjørnstad, Ragnhild Aaløkken, June Åsheim, Arvind Y. M. Sundaram, Caroline N. Felde, G. Henriette Østby, Marianne Dalland, Wenche Sjursen, Christian Carrizosa, Magnus D. Vigeland, Hanne S. Sorte, Ying Sheng, Sarah L. Ariansen, Eli Marie Grindedal, Gregor D. Gilfillan

**Affiliations:** 1 Department Medical Genetics, Oslo University Hospital and University of Oslo, Oslo, Norway; 2 Department of Clinical & Molecular Medicine, NTNU and Department of Medical Genetics, St Olavs Hospital, Trondheim, Norway; 3 Present Address: Department of Forensic Sciences, Oslo University Hospital, 0372 Oslo, Norway

**Keywords:** Cancer genetics, Cancer genomics, Medical genomics, Genomic analysis, Next-generation sequencing

Correction to: *European Journal of Human Genetics* 10.1038/s41431-023-01494-7, published online 29 November 2023

In this article, the wrong figure appeared as Fig. 3B due to typesetting mistake.; the Fig. 3 should have appeared as shown below.

**Figure:**
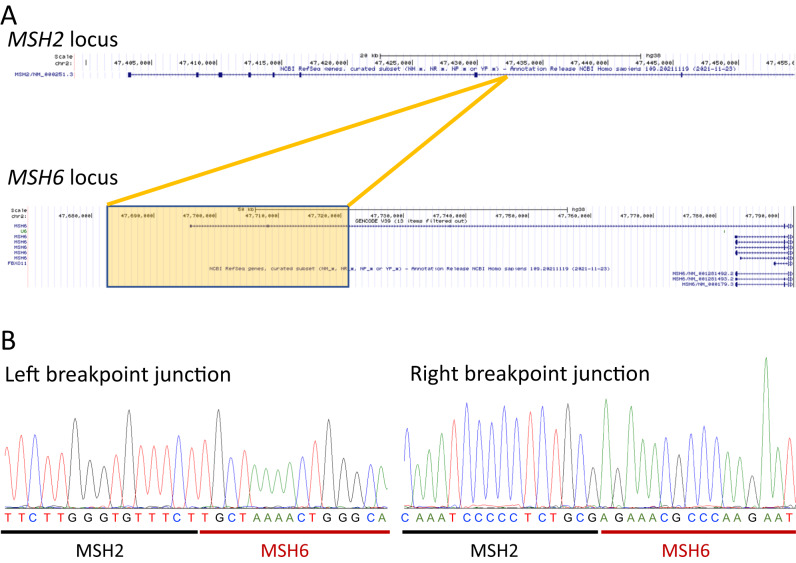

The original article has been corrected.

