# Supplementary material for: A 39 kb structural variant causing Lynch Syndrome detected by optical genome mapping and nanopore sequencing
Source: Eur J Hum Genet. 2023 Nov 29;32(5):513–20. doi: 10.1038/s41431-023-01494-7 (PMC11061271; doi:10.1038/s41431-023-01494-7)
Supplement: Supplementary file 1 — Supplementary Figures [file 41431_2023_1494_MOESM1_ESM.pdf]

Supplementary Fig. 1

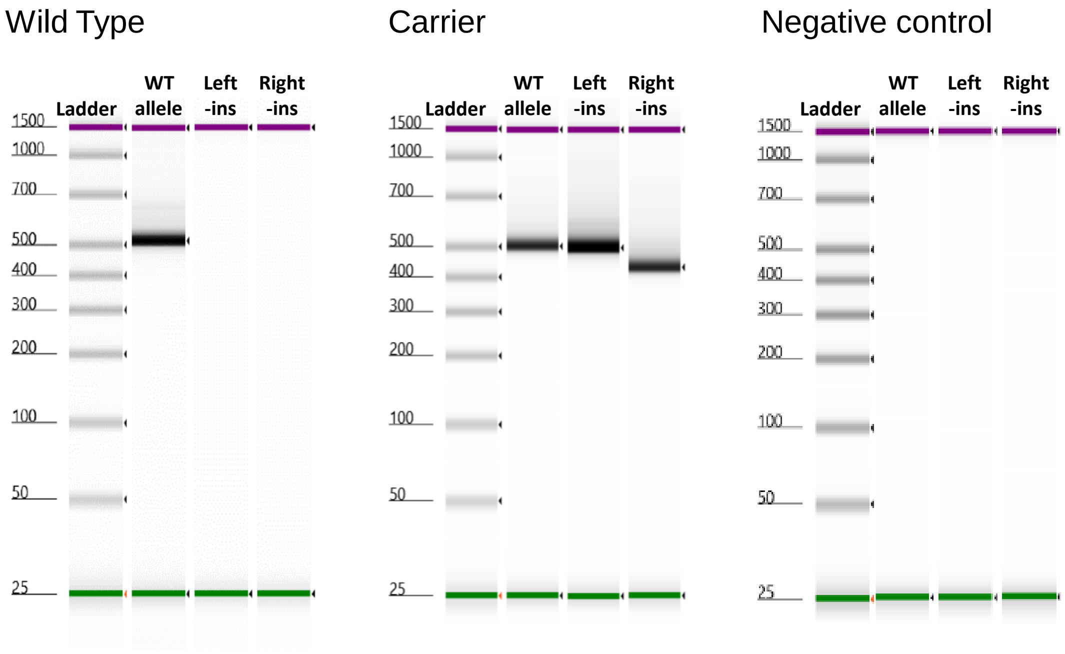

Supplementary Fig. 1

PCR confirmation of insertion junctions. Three PCR amplicons were designed to amplify i. the wild-type MSH2 sequence flanking the insertion point, ii. The left-hand insertion junction of *MSH2-MSH6* sequence and iii. The right-hand junction of *MSH6-MSH2* sequence. Successful amplification confirmed the identity of the inserted sequence. These PCR reactions were then used to screen additional individuals for carrier status of the insertion, as exemplified here. Amplified PCR products were run on a Tapestation 4200 with DNA 1000 reagents. Left panel shows amplification only from the wt sequence in an unaffected individual. Middle panel shows amplification of all three PCR products in a carrier. Right panel shows no-template control reactions. For primer sequences, see manuscript Materials and Methods, subsection “Breakpoint PCR and Sanger sequencing”.

Supplementary Fig. 2

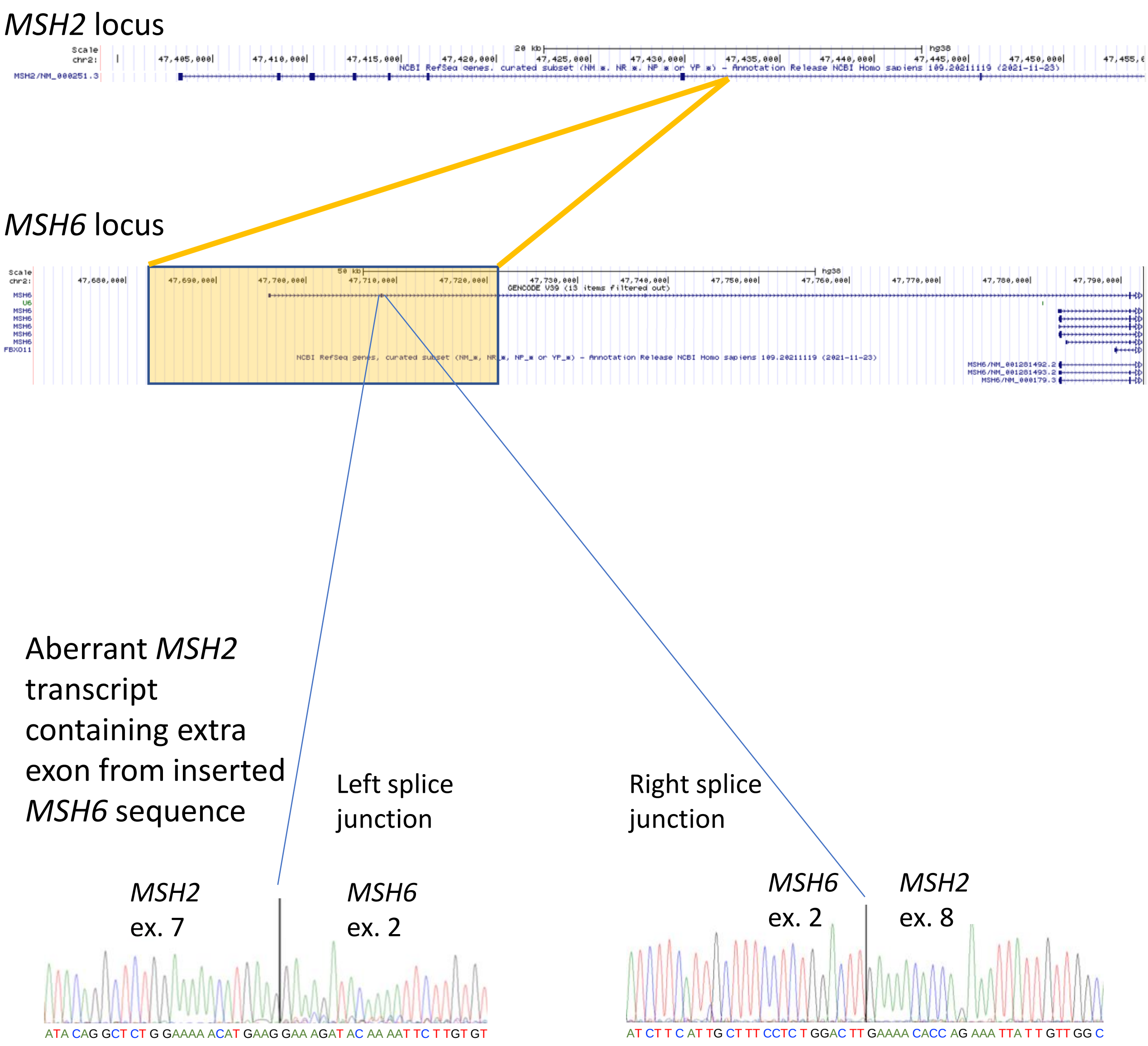

Supplementary Fig. 2

Aberrant *MSH2* transcript containing *MSH6* exon. Figure shows genomic insertion of *MSH6* sequence (orange box in middle image, genome-browser view) into *MSH2* locus (top image, genome browser view). Bottom images are Sanger sequencing traces of aberrant transcript amplified by PCR, showing left and right splice junctions of *MSH6* exon 2 spliced between exons 7 and 8 of *MSH2*. For primer sequences and combinations, see Table 1 in manuscript.

Supplementary Figure 3

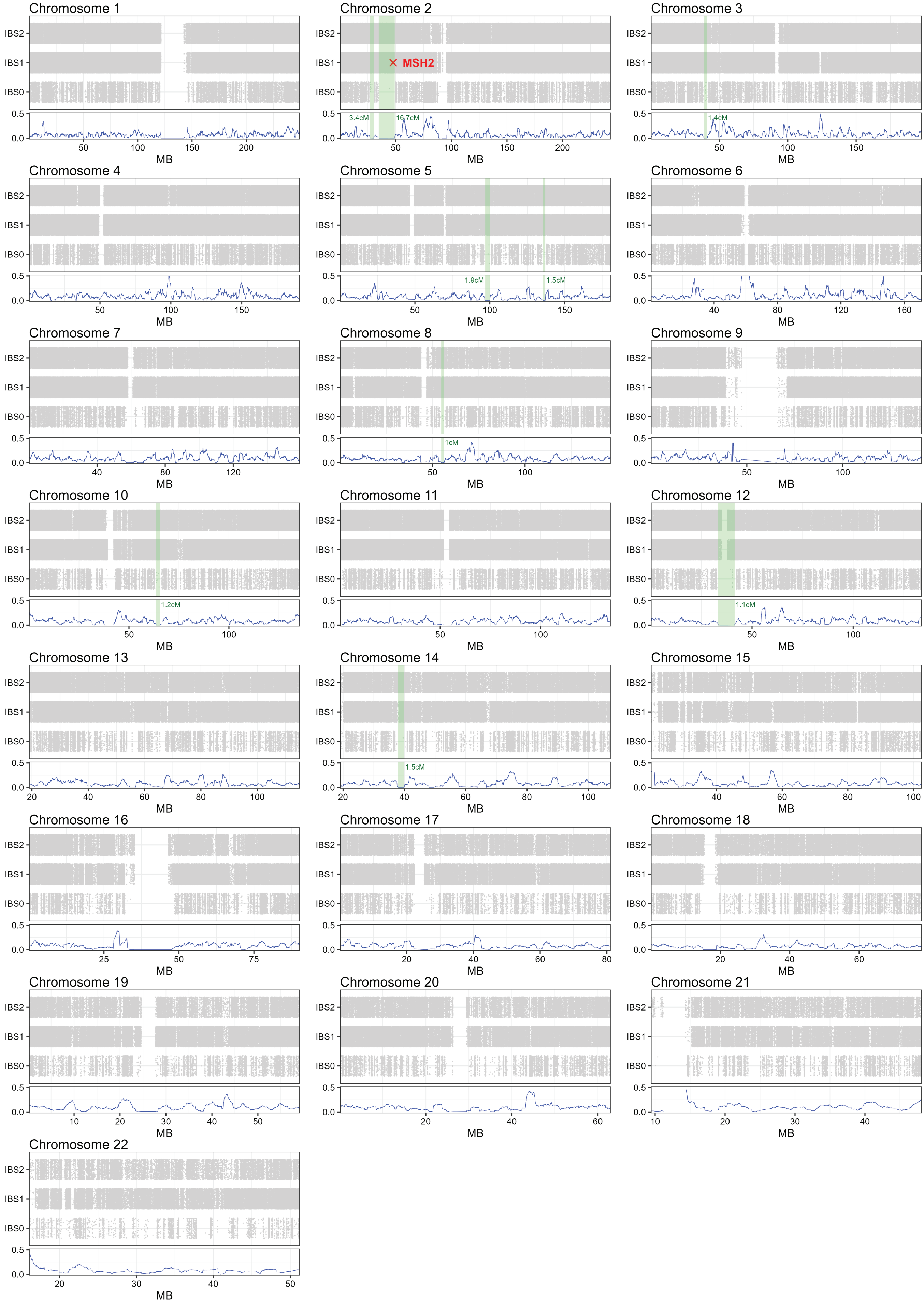

Supplementary Fig. 3

Chromosomal Identity by State plots. Each dot represents a variant, showing its physical position along the chromosome and the number of alleles shared between the index patients (i.e., IBS state, either 0, 1 or 2). A vertical jitter was added to reduce overplotting. The blue curves below each chromosome show a rolling average of $w = I(IBS = 0)$, as explained in Methods. Regions called as IBD segments are highlighted in green. The location of the *MSH2* gene is marked in red.
